# Predicting 3D Structures of Lasso Peptides

**DOI:** 10.21203/rs.3.rs-4579522/v1

**Published:** 2025-04-01

**Authors:** Zhongyue Yang, Xingyu Ouyang, Xinchun Ran, Han Xu, Yi-Lei Zhao, A. Link, Runeem Al-Abssi

## Abstract

Lasso peptides (LaPs), characterized by their entangled slipknot-like structures, are a large class of ribosomally synthesized and post-translationally modified peptides (RiPPs), with examples functioning as antibiotics, enzyme inhibitors, and molecular switches. Despite thousands of LaP sequences predicted by bioinformatics, only around 50 distinct LaPs have been structurally characterized in the past 30 years. Existing computational tools, such as AlphaFold2, AlphaFold3 and ESMfold, fail to accurately predict LaP structures due to their irregular scaffold featuring a lariat knot-like fold and the presence of an isopeptide bond. To address this challenge, we developed LassoPred, designed with a classifier to annotate the ring, loop, and tail of an LaP sequence and a constructor to build a 3D structure. Leveraging LassoPred, we predicted 3D structures for 4,749 unique LaP core sequences, creating the largest *in silico* -predicted lasso peptide structure database to date. LassoPred is publicly available through a web interface (https://lassopred.accre.vanderbilt.edu/) and a command-line tool, supporting future structure-function relationship studies and aiding in the discovery of functional lasso peptides for chemical and biomedical applications.

### 1. Introduction

Lasso peptides (LaPs) are a class of ribosomally synthesized and post-translationally modified peptides (RiPPs) defined by their threaded structure, formally a [1]rotaxane [1]. This structure consists of a macrolactam ring, formed by an isopeptide bond between the N-terminal α-amino group and the side chain carboxyl group of an aspartate or glutamate residue[2]. The ring encircles the C-terminal tail of the peptide, forming a distinct right-handed lariat knot-like structure[3], with the residues above and below the plane of the ring referred to as the "upper plug" and "lower plug", respectively (Figure S1). The terminology "plug" does not have an implication on its mechanical role in holding the ring in place. These plug residues can come from a wide variety of amino acids—17 out of 20 for the upper plug and 14 out of 20 for the lower plug (Figure S2). The "plug position" indicate the two residues between which the ring is located based on the PDB structure, rather than which two are mechanically locking the peptide. Sterically bulky residues, which are responsible for preventing the ring from unthreading, can be located a few amino acids away from the plugs. They impart LaPs with high thermal stability and resistance to protease degradation. LaPs exhibit biological relevance as antibiotics[4-7], enzyme inhibitors[8, 9], and receptor antagonists[10, 11]. Lasso peptides also demonstrate chemical functions as dynamic covalent materials[12] and thermally-actuated switches[13, 14].

Despite sharing the lasso structure, LaPs demonstrate considerable structural variability due to different ring, loop, and tail size as well as highly variable sequences. Our knowledge into the full structural diversity of LaPs is highly limited, hindering the prioritized discovery of functional LaPs for applications. Until March 15$^{th}$ 2024, only 47 unique LaP structures have been experimentally determined and deposited in the Protein Data Bank (PDB) (Table S1). However, over 4,000 putative unique LaP sequences are identified through genome-mining algorithms such as RODEO [15-17] (see Supporting Information, Data.csv), thereby presenting a huge knowledge gap for three-dimensional (3D) structural prediction and characterization.

Given that LaPs are relatively short and possess irregular scaffolds, AlphaFold2[18] and ESMFold[19] fail to model the lariat knot-like topology of lasso peptides (LaPs) (Figure S3). AlphaFold3[20], however, shows capability in predicting lasso peptide structures, as evidenced by its ability to reproduce the 3D structures of 79% of LaPs in the PDB (e.g., stlassin, Figure 1A, 1B and Table S1). This is likely due to AlphaFold3's improved framework, which reduces hallucinations and enhances the accuracy of coordinate generation. [20]. Despite this improvement, AlphaFold3 exhibits poor generalizability. Tested with a manually curated dataset of 12 LaPs with known structural annotations reported in the literature but undeposited in the PDB [21-28] (except for capistruin, Table S2), AlphaFold3 failed in 10 out of 12 LaPs (87%, Figure 1C, 1D, and Table S2). Of the remaining two, AlphaFold3 correctly predicted the lariat-knot topology of capistruin and mycetohabin-15, although the plug annotation for the latter is incorrect (Table S2, Figure 1C and 1D). On a more challenging dataset, we curated 40 randomly selected RODEO-mined LaP sequences with unknown annotations and structures. In this test, AlphaFold3 predicted only three sequences to adopt a lariat-knot topology (success rate: 8%, Figure 1E, 1F, and Table S3), and their annotation accuracy remains experimentally undetermined. In most cases, AlphaFold3 (AF3) incorrectly predicts LaPs to adopt linear ($^{LaP}$Test1), cyclic ($^{LaP}$Test2), branched cyclic ($^{LaP}$Test3), and helical ($^{LaP}$Test4- $^{LaP}$Test5) structures (Figure 1E and Table S4). The low success rate of AF3 in predicting lasso-fold proteins not present in the PDB highlights its difficulty with extrapolation, similar to challenges observed when predicting fold-switched protein conformations[29], posing a major roadblock to the community[30-32].

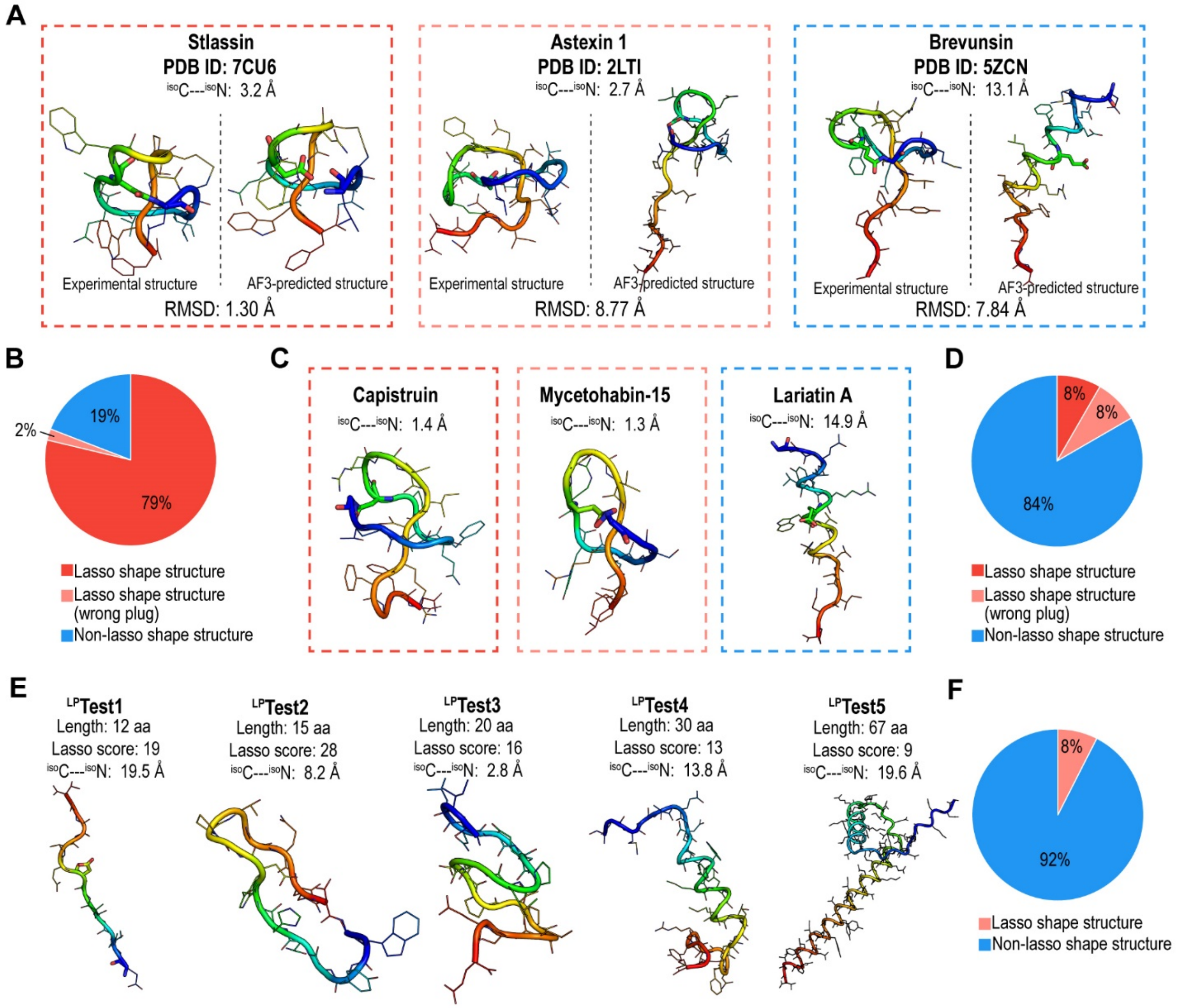

**Figure 1**. AF3 performance in predicting LaP structures. (A) AF3 predictions on known LaP structures, with experimentally determined structures display in each group, RMSD were calculated on Cα atoms. The structure ribbon is colored from blue at the N-terminus to red at the C-terminus. Isopeptide $^{iso}$C---$^{iso}$N distances were measured between the N atom of the N-terminus residue and the C atom of the carboxyl group in the isopeptide donor residues (Asp or Glu in the 7$^{th}$/8$^{th}$/9$^{th}$ position). The dashed frames indicate the classification color pattern used in panel (B). (B) Pie chart showing the performance of AF3 on PDB-deposited LaPs, with predicted structures classified into three categories. A predicted structure is classified as having a "lasso fold" if the wrapping is observed with a C–N bond distance of less than 4.0 Å. (C) AF3 predictions for 12 LaPs with a known plug position but not deposited in the PDB. (D) Pie chart showing the performance of AF3 on LaPs with known plug position but not deposited PDB structures, with predicted structures classified into three categories, using the same color pattern as in panel (A). (E) AF3 predictions for LaPs with unknown structures. The illustrated LaPs were randomly selected from the RODEO dataset, employing stratified sampling based on total sequence length. A more comprehensive test involving 40 randomly-selected lasso peptide sequences is shown in Table S3. Lasso scores refer to the potential of peptide sequences to be classified as lasso peptides, predicted from RODEO. The sequences for the representative LaPs are available in Table S4. (F) Pie chart showing the structure prediction results corresponding to Table S3, with predicted structures classified into two categories (lasso shape and non-lasso shape), as the true plug positions are unknown.

Here we developed LassoPred for lasso peptide structure prediction. LassoPred involves an annotator-constructor architecture, where the annotator predicts up to three sets of sequence annotations, and the constructor converts each predicted sequence annotation to a 3D lariat-like structure. LassoPred's generalizability was evaluated using a "blind test" consisting of 12 LaPs (Figure 1C and 1D). These sequences have less than 60% sequence identity or similarity compared to any of the LaP sequences used in the training and test sets. Using LassoPred, we built 4,749 distinct LaP structures that were predicted using genome mining analyses by RODEO curated on March 15$^{th}$, 2024 (Supporting Information, Data.csv). To allow public access to the database and the prediction tool, we set up a web interface (https://lassopred.accre.vanderbilt.edu/). Besides advancing knowledge of lasso peptide structural diversity, LassoPred will facilitate NMR solution structural determination and the discovery of functional lasso peptides for therapeutic and industrial applications, aiding in the development of treatments for infectious diseases and enzyme inhibitors.

### 2. Results

LassoPred is designed to translate lasso peptide sequences into 3D structures. It comprises two modules: an annotator that predicts three distinct sets of sequence region annotations (i.e., length of ring, loop, and tail) for an input sequence, and a constructor that builds 3D LaP structures based on predicted sequence annotations (Scheme 1). With an input sequence, the annotator first decomposes the sequence into overlapping dipeptide fragments, then leverages two support vector classifiers: an isopeptide classifier and a plug classifier, to predict the dipeptide fragments that contain the isopeptide-donating residue and plug residues, respectively, and eventually generate three sets of possible sequence annotations that share the same ring length but various loop/tail length. Each set of sequence annotation will convert to a 3D LaP structure using the constructor, which consists of upgraded modules from LassoHTP [33] to build the lariat-like LaP scaffold, generate LaP mutants, and optimize LaP structures based on molecular mechanics (see Computational Methods).

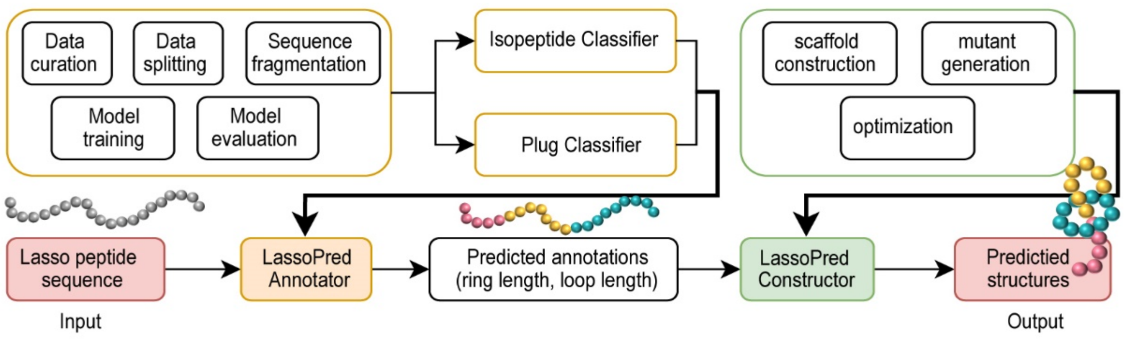

**Scheme 1**. The design architecture of LassoPred. Taking a lasso peptide sequence as input, LassoPred first uses its annotator to predict up to three sets of sequence annotations (ring, loop, tail length), and then employs its constructor to transform each set of annotations into a 3D structure. The annotator consists of an isopeptide classifier and a plug classifier, each trained as a machine learning classifier using 47 lasso peptide PDB structures. The constructor includes a scaffold construction module to build an all-alanine lasso peptide scaffold template matching the annotated ring, loop, and tail lengths, a mutant generation module to create a 3D lasso peptide structure matching the input sequence, and an optimization module to refine the generated structures using a molecular mechanics force field.

### 2.1 Development of LassoPred's Annotator

The primary challenge in building the LassoPred annotator was the small dataset, as there are only 47 lasso peptide structures available in the PDB (Table S5). This small dataset poses a significant problem for the generalizability of the model due to the potential bias in test set performance, and the risk of overfitting. To systematically address these issues, we proposed three strategies. First, we employed data augmentation through peptide fragmentation, enriching the dataset by breaking down each LaP sequence into smaller peptide fragments for model training. Both the isopeptide and plug classifiers were trained separately to assign each fragment to its correct sequence region and subsequently reconstruct the isopeptide and plug positions from the predicted fragments. Second, we used transfer learning by incorporating pre-trained embeddings from protein language model (ESM2 and AF2 [18, 19]), which introduced evolutionary information into the model features. Third, to minimize performance bias and overfitting, we used repeated holdout validation to evaluate all performance metrics [34]. This involves conducting 100 training/test set splits at a 4:1 ratio with stratified sampling to ensure that the test set maintains a consistent distribution of ring and loop lengths with the overall dataset (splits_100.csv). Machine learning (ML) algorithms and their hyperparameters were selected based on the average model performance across these splits. These methods are designed to enhance the robustness of the model and reduce the impact of the small dataset on generalizability. Below, we will discuss the implementation and benchmarking of these strategies to develop the isopeptide and plug classifiers underlying the LassoPred annotator.

To develop the isopeptide classifier, we first converted each sequence of N amino acids into N overlapping peptide fragments, resulting in a total of 905 fragments. We then labeled each dipeptide fragment as '0', '1', or '2', where '1' indicates residues within the ring, '0' marks the ring-loop boundary (i.e., the position of the isopeptide-donating residue), and '2' corresponds to residues in the loop or tail. Since the isopeptide-donating residue must be Glu or Asp that is located at the 7$^{th}$, 8$^{th}$, or 9$^{th}$ positions (prior knowledge derived from all known lasso peptide structures), we leveraged only the peptide fragments containing residues between the 6$^{th}$ and 10$^{th}$ position of each LaP sequence for training the isopeptide classifier (Figure 2A). Using dipeptide fragments and ESM2 L33 embedding, the isopeptide classifier achieves strong predictive performance in classifying the peptide fragments into one of the three categories, featuring a ROC AUC of 0.97±0.03, a fragment classification accuracy of 0.91±0.06, and a fragment classification F1 score of 0.90±0.08 across 100 splits. The reconstruction process determines the isopeptide position by identifying the dipeptide fragment with the highest likelihood of being labeled as '0' (the ring-loop boundary). As a result, the isopeptide classifier achieves a nearly perfect accuracy for identifying the isopeptide-donating residue (accuracy: 1.00±0.02), with only 1 out of 100 splits failing to reach 100% accuracy. The high accuracy of the isopeptide classifier is expected because the isopeptide position can be identified merely by finding Glu or Asp located at the 7$^{th}$, 8$^{th}$, or 9$^{th}$

positions in 42 out of the 47 LaP sequences curated from PDB, giving a based-line accuracy of 0.89.

Developing the plug classifier is much more challenging due to the wider variety of amino acid types that can act as the plug (17 out of 20 for the upper plug and 14 out of 20 for the lower plug, Figure S2) and the broader distribution of loop length (3-20 amino acids), as observed from the 47 LaP structures in the PDB. Similar to the isopeptide classifier, we labeled each peptide fragment as '0', '1', or '2', where '1' indicates fragments within the loop, '0' marks the loop-tail boundary (considering both upper and lower plug residues), and '2' corresponds to residues within the tail (Figure 2A). Given the complexity of developing the plug classifier, we began by benchmarking several fragmentation strategies: dipeptide, tripeptide, and tetrapeptide fragmentations, as well as dipeptide and tripeptide fragmentations with a truncated ring sequence. Each set of the benchmarking employs ESM embedding and a grid search over a combination of machine learning algorithms and hyperparameters (Table S6), with the model performance evaluated across 100 splits (Table S7). Each of these fragmentation strategies corresponds to a specific approach for reconstructing the plug positions from the predicted fragments (detailed in the Text S1 and Table S8-S9). Eventually, ring-truncated dipeptide fragmentation demonstrates the best predictive performance in identifying the correct plug position from one prediction (top 1 accuracy: 0.60±0.15) or three predictions (top 3 accuracy: 0.85±0.10, Table S7). Although using longer fragments, such as tetrapeptides, improves the accuracy of categorizing fragments into the three classes (Class 0, 1, 2), featured by a ROC AUC score of 0.96±0.02 compared to 0.91±0.04 for ring-truncated dipeptides fragments (Table S7), the performance in reconstructing the plug position is weaker (top 3 accuracy: 0.79±0.12 vs. 0.85±0.10, Table S7). This is likely due to the information loss when attempting to reconstruct the plug position from multiple fragments that are predicted with a label of '0'. Additionally, compared to dipeptide fragmentation, ring-truncated dipeptide fragmentation shows better plug prediction accuracy (top 3 accuracy: 0.85±0.10 vs. 0.80±0.10, Table S7) because it removes the ring sequence predicted by the isopeptide classifier and focuses the model on the loop and tail regions. This minimizes noise caused by irrelevant sequence information.

With the dipeptide fragmentation strategy, we tested eight feature engineering approaches, based on embeddings from protein language models ESM2, AF2, and SaProt (a new model that combines residue sequences with 3D structural information through a structure-aware vocabulary[35]). For each test, we applied four different ML models, including Random Forest Classifier (RFC), K-Neighbors Classifier (KNC), Gradient Boosting Classifier (GBC), and Support Vector Classifier (SVC), which are identical to those used in the fragmentation test. The hyperparameters for each model were optimized through grid search (Table S6), and the model that achieves the highest plug annotation accuracy (i.e., top 1-3 accuracy) is used in the performance statistics (Figure 2B). Based on repeated holdout testing across 100 splits, the 33$^{rd}$ layer of the "ESM2_t33_650M" model (ESM2 L33) shows the best performance, achieving an overall ROC AUC of 0.91±0.04, a Top 3 accuracy of 0.85±0.10, and Top 3 F1 score of 0.91±0.06 (Table S10).

To minimize bias, we selected the ML algorithm and hyperparameters for the plug classifier that showed the minimal deviation from the mean performance across all metrics from the 100 splits modeled using ring-truncated dipeptide fragmentation and ESM2 L33 embedding. Specifically, this refers to the split with the minimum absolute error from the mean across various performance metrics, including fragment classification accuracy, fragment classification F1 score, one-vs-rest weighted ROC AUC, ROC AUC for Class 0, Class 1, and Class 2, as well as top 1, top

2, and top 3 accuracy (Table S10). On this split, the support vector classifier model, whose hyperparameters are optimized with grid search, demonstrates the best predictive accuracy (Table S11-S12), largely owing to its margin-based approach and adaptability to high-dimensional embeddings. This selected split's test set exhibits a similar distribution to the overall dataset in terms of loop length (test set: 5.8±2.4 aa vs. overall: 6.4±3.5 aa, Figure 2D) and total length (18.5±2.8 aa vs. 19.3±3.9 aa, Figure 2C).

Based on this test set (10 LaPs, Table S11), the plug classifier demonstrates strong performance in classifying the dipeptide fragments into the correct sequence region (i.e., Class 0, 1, and 2). This is reflected in its class 0 ROC-AUC score of 0.87 (Figure 2F), a one-vs-rest weighted ROC-AUC score of 0.90 (Table S13), fragment classification accuracy of 0.81 (Table S13), and fragment classification F1 score of 0.81 (Table S13). In annotating the correct plug position, the plug classifier achieves accuracies of 0.60, 0.80, and 0.90 for the top 1, 2, and 3 predictions, respectively (Table S13). In the test set, the only LaP that is missed in the top 3 predictions is benenodin-1, which exists in two isomeric states (PDB IDs: 5TJ1 and 6B5W), with one state placed in the training set and the other in the test set. On the other hand, the isopeptide classifier exhibits perfect accuracy in identifying the isopeptide-donating residue (accuracy: 1.00) and strong performance in classifying dipeptide fragments into the three regions, with a class 0 ROC-AUC score of 1.00, a one-vs-rest weighted ROC-AUC score of 0.98, and a fragment classification accuracy and F1 score of 0.90 (Figure 2E, Table S13). Using the top predicted location for the isopeptide-donating residue (the isopeptide classifier) and the top three predicted locations for the upper plug (the plug classifier), the LassoPred annotator generates up to three sets of sequence annotations per input. For LaPs with shorter sequences (e.g., those under 15 amino acids), the annotator may produce only one or two sets of sequence annotations.

To assess the model's stability, we performed a data splitting test using ring-truncated dipeptide fragmentation, ESM2 L33 embedding, and the SVC model (Figure 2G and Table S14). Under each training/test splitting ratio, the performance metrics were evaluated using repeated holdout validation across 100 splits. As the training set size decreased from 83% (39 LaPs) to 74% (35 LaPs), and further to 49% (23 LaPs), the model's Top 3, 2, and 1 accuracy remained stable, ranging from 0.86±0.11 to 0.83±0.08 for Top 3 accuracy, from 0.72±0.08 to 0.76±0.14 for Top 2 accuracy and from 0.58±0.15 to 0.57±0.09 for Top 1 accuracy (Table S14). This shows that even with a reduced percentage training data, the model's predictive performance remains stable.

Notably, the current dataset includes sequences that differ by only a few mutations, reflecting the limited sequence variation of lasso peptides. These similar sequences were included to ensure consistent plug position predictions despite mutations, though this could raise a concern about potential data leakage. To address this concern, we tested the model on a cleaned dataset of 36 LaPs with <80% sequence similarity, determined by multiple sequence alignment (calculated as matched residues in global alignments. Using ring-truncated dipeptide fragmentation, ESM2_L33 embedding, the SVC model, and a 4:1 training/test split (29 in the training and 7 in the test), the model's performance remains similar for the top 3 prediction accuracy (original: 0.85±0.10 vs. clean: 0.86±0.13) and the one-versus-rest-weighted ROC AUC (original: 0.92±0.04 vs. clean: 0.88±0.05), but drops for the Top 1 (original: 0.63±0.13 vs. clean: 0.49±0.18) and Top 2 accuracy (original: 0.75±0.13 vs. clean: 0.69±0.19, Figure 2H and Table S15). This shows that the presence of mutant sequence in the dataset does not affect the model's ability to achieve at least one correct annotation in the top 3 predictions.

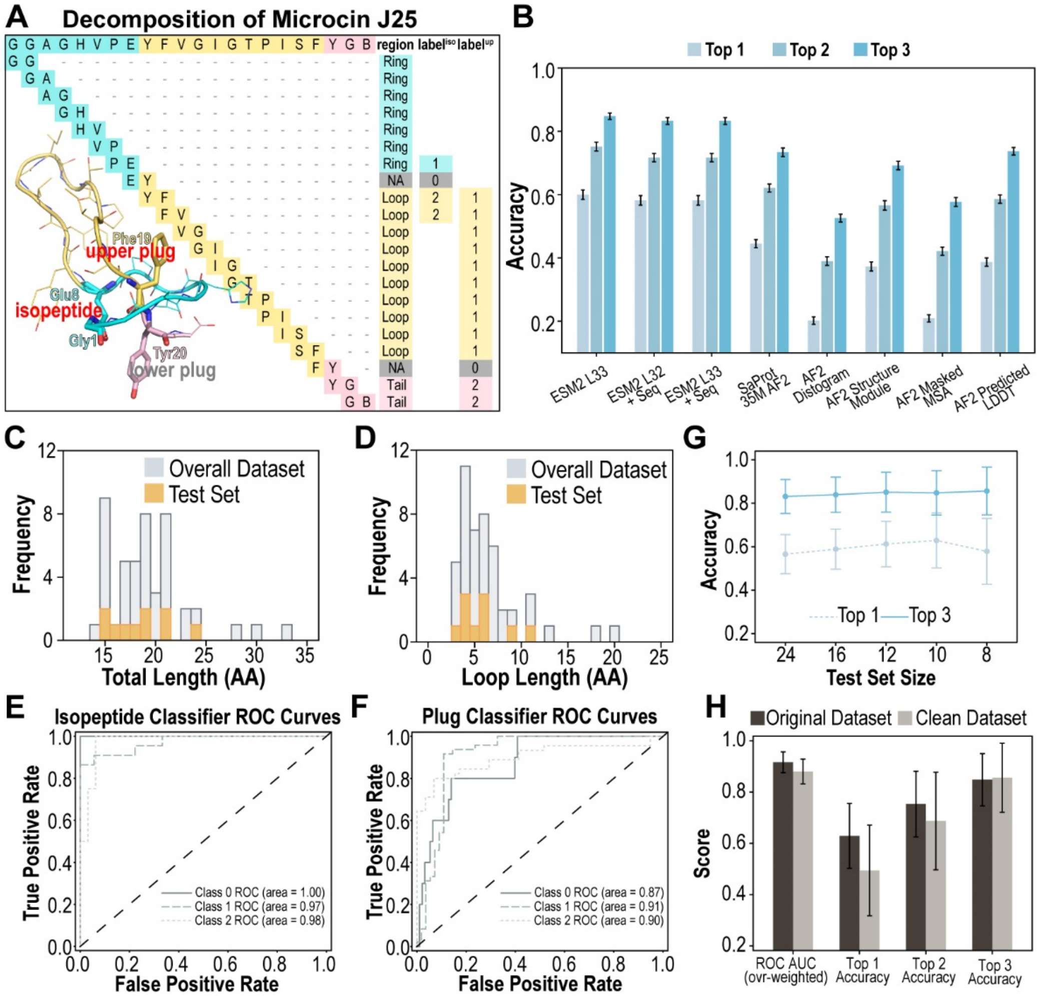

**Figure 2.** Performance of LassoPred's annotator. (A) Microcin J25 serves as an example of sequence splitting, with the sequence divided into three parts: ring (cyan), loop (yellow), and tail (pink). The sequence is split into consecutive dipeptide fragments, each overlapping with its neighbors by one amino acid. The categories of the fragments are labeled in the right column, with boundary fragments marked as "NA". The accompanying cartoon and stick structure highlight the isopeptide and plug residues in Microcin J25, following the same color scheme as sequence. (B) Comparison of plug prediction performance using various sequence featurization methods through repeated holdout validation. Specific information about each featurization method is described in Table S10. Using dipeptide fragmentation on ring-truncated sequences, each of the 100 splits was tested with four different ML models: random forest classifier (RFC), K-neighbors classifier (KNC), gradient boosting classifier (GBC), and support vector classifier (SVC). The hyperparameters for each model were optimized through grid search, and the model that achieves the highest accuracy is used in the performance statistics. Bars represent the mean values, with standard deviations shown as error bars. (C) and (D): Distribution of the sequence length and loop length, respectively, for the entire dataset (47 LaPs, grey) and the selected split (10 LaPs, orange). (E) and (F): ROC curves for the isopeptide and plug classifier, respectively. For the isopeptide classifier, Class 0, 1, and 2 correspond to the isopeptide, ring, and loop regions, respectively. For the plug classifier, these classes refer to the plug, loop, and tail regions, respectively. (G) Data splitting test for plug prediction accuracy using ring-truncated dipeptide fragmentation, ESM2_L33 embedding, and the SVC model with optimized hyperparameters on the selected holdout set. For each splitting ratio, Top 1 and Top 3 accuracy were assessed via repeated holdout validation of 100 splits, applying a 4:1 training-to-test ratio and stratified sampling. Accuracy is represented by mean values, with standard error shown as error bars, evaluating the variation of the average performance across different test set ratios. (H) Model performance comparison of the original and clean dataset using ring-truncated dipeptide fragmentation, ESM2_L33 embedding, and the SVC model with optimized hyperparameters on the selected holdout set. The clean dataset, defined by sequence similarity of less than 80%, contains 36 LaP sequences in total. For both datasets, all performance metrics were evaluated using repeated holdout validation across 100 splits, applying a 4:1 training-to-test ratio and stratified sampling.

Finally, to rigorously assess the generalizability of the annotator, we conducted a "blind test" using a curated set of 12 distinct LaPs from the literature [5, 21-24, 36] (Table 1). Their sequence annotations have been confirmed by solution NMR spectroscopy experiments or biochemical assays, but they are not deposited in the PDB except for capistruin that has a co-crystalized structure with RNA polymerase. This blind test is particularly challenging, as all LaP sequences exhibit low similarity (≤0.65) and identity (≤0.46) with the dataset of 47 LaPs curated from PDB. In this test, LassoPred's annotator achieves top 1, 2, and 3 prediction accuracies of 0.58, 0.67, and 0.92, respectively, consistent with the performance observed on the hold-out test set (Table 1). The isopeptide classifier predicts all isopeptide-donating residues with 100% accuracy, so the annotator's prediction accuracy depends solely on the performance of plug classifier. One notable example is caulonodin VI, the longest LaP in the test set with 19 amino acids residues and multiple bulky residues in the loop/tail region (e.g., Lys, Arg, Gln, and Tyr) that could potentially serve as plugs. Despite the complexity, LassoPred accurately identifies the correct plug position on its first guess. Additionally, the annotator gives an identical set of predicted annotations for RES-701-1 vs. RES-701-3, indicating the insensitivity of prediction results to point mutations. The predictive performance of LassoPred in the "blind test" highlights the robustness and generalizability of the model beyond the training data and the holdout test, supporting its ability to deliver reliable sequence annotations, which are crucial for the constructor to build accurate 3D structures.

**Table 1**. Performance of annotation prediction on the blind set. The results for the isopeptide prediction are not shown because it correctly predicts isopeptide-donating residues with 100% accuracy. The top three predicted upper plugs from the plug classifier are shown, with the correct one in bold. Similarity and identity were compared with the 47 lasso peptides in the dataset, and the maximum values are presented. The isopeptide-donating residue and plug residues are indicated in bold font in the sequence.

| Lasso Peptide Name | Sequence | Max Similarity/ Identity | Real upper plug | Top 1 upper plug | Top 2 upper plug | Top 3 upper plug |
|---|---|---|---|---|---|---|
| RES-701-1 | GNWHGTAP**D**WFF**N** YYW | 0.50/0.30 | 13 | 14 | 12 | **13** |
| RES-701-3 | GNWHGTSP**D**WFF**N** YYW | 0.50/0.30 | 13 | 14 | 12 | **13** |
| capistruin | GTPGFQTP**D**ARVI**S**R FGFN | 0.53/0.33 | 14 | 16 | **14** | 13 |
| Lariatin A | GSQLVYR**E**WVG**H**S NVIKP | 0.44/0.26 | 12 | **12** | 14 | 13 |
| Fuscanodin | WYTAEWGL**E**LIFVF P**R**EI | 0.44/0.28 | 16 | **16** | 15 | 14 |
| Cellulonodin-1 | WIQGKWGL**E**IYLIFP **R**YL | 0.44/0.26 | 16 | **16** | 15 | 14 |
| Cellulonodin-2 | GAADDDTA**D**MNTA **K**YY | 0.44/0.24 | 14 | **14** | 12 | 13 |
| Burhizin | GGAGQYK**E**VEAG**R** WSDRIDSDDE | 0.57/0.31 | 13 | 16 | 14 | 21 |
| Mycetohabin-15 | GGSGQYR**E**AGVG**R**F L | 0.53/0.32 | 13 | **13** | 11 | 12 |
| Mycetohabin-16 | GGSGKYK**E**AGVG**R**F LD | 0.56/0.36 | 13 | **13** | 11 | 14 |
| caulonodin IV | SFDVGTIK**E**GLVSQY **Y**FA | 0.56/0.34 | 16 | 15 | 14 | **16** |
| caulonodin VI | AGTGVLLP**E**NTQIK **R**YDPA | 0.47/0.27 | 15 | **15** | 16 | 14 |
| Accuracy | | | | 0.58 | 0.67 | 0.92 |

### 2.2 Development of LassoPred's constructor

Using predicted sequence annotations (ring, loop, and tail lengths) as input, the constructor first builds an all-alanine LaP scaffold matching the given annotations, mutates side chains to match the input sequence, and then optimizes the structure with molecular mechanics force field in AMBER. Users can optionally include MD sampling to generate a conformational ensemble, allowing for clustering analysis. To develop LassoPred's constructor, we substantially innovated several core modules of LassoHTP. First, we expanded the scaffold library to accommodate a wider range of loop lengths, increasing from the original 3–20 to 3–50 amino acids residues (Figure

S4), enabling the construction of more diverse LaP structures. Second, we replaced the scaffold creation engine to accelerate the molecular model building, reducing construction time from approximately 2 hours using steered MD in LassoHTP to less than 10 minutes with SWISS-MODEL[37]. Third, we implemented critical functionalities that do not exist in LassoHTP, including left-handed lasso peptide construction, PyMOL-interfaced mutant generation, and conformational clustering of MD trajectories (described in the Computational Methods section). Finally, to enhance the robustness of the constructor, the force field file of the isopeptide bond, including atomic labels and parameters, was formatted to ensure full compatibility with the canonical amino acids in the scaffold.

By integrating the annotator and constructor, LassoPred works as an automatic pipeline in Python or Shell. For an input lasso peptide sequence, the script first checks if the sequence is eligible for prediction by verifying three conditions: whether a potential isopeptide-donating residue (Asp or Glu) exists at the $7^{th}$, $8^{th}$, or $9^{th}$ position of the sequence, whether the sequence consists of recognizable amino acid letters, and whether the length is appropriate (i.e., total length $\geq$ 12 aa, and the distance from the last amino acid to the first estimated isopeptide is at least 5 aa, given that the minimum loop length and tail length are 3 and 2, respectively). If all these conditions are met, LassoPred will leverage the annotator to predict up to three sets of annotations and the constructor to predict a 3D LaP structure for each set of annotation. The total duration for sequence annotation, structural construction, and structural optimization typically takes less than 5 minutes on EVGA GeForce RTX 2080, allowing an efficient sequence-to-structure conversion for lasso peptide design and engineering. MD sampling is optional for users and can be customized according to local computing resources and specific needs.

#### 2.3 Assessment of LassoPred for Structural Determination

To assess LassoPred's accuracy for structural determination, we assessed how closely LassoPred's constructed 3D structures matches their respective PDB structure in the test set of selected split (Table S11). LassoPred identifies the correct annotation among its top 3 predictions in 9 out of 10 LaPs (Table S16), failing to predict the isomeric state 1 of benenodin-1 (PDB ID: 5TJ1), which converts to the isomeric state 2 (PDB ID: 6B5W) at various temperatures [13]. The 10 LaPs in the test set undergo structural construction and optimization using LassoPred, with 9 being correctly annotated and $^{test}$V7 (benenodin-1) having the top 1 predicted annotation. Referencing to the PDB structure, 8 optimized LaP structures involve an $C_\alpha$ RMSD value lower than 4.0 Å—a numerical cutoff to determine quality of predicted structures [38]. This 4.0 Å threshold is further justified by a 30 ns classical MD simulation of the linear-core peptide of microcin J25 (random coil state), during which none of the sampled snapshots fall within 4 Å RMSD compared to the PDB reference (Figure S5C).

The average RMSD of the 9 optimized LaP structures shown in Figure 3A, all of which have correct annotations, is 3.2±1.0 Å for all Cα atoms and 1.0±0.6 Å for the Cα atoms of local interlocked structural moiety that consists of the isopeptide-donating residue, plug residues, along with their adjacent residues (Table S17). $^{test}$V7 was excluded from this calculation because it involves incorrect annotations in all three prediction trials. However, even by including predicted structures with an incorrect annotation, the average RMSD is still 3.2±0.9 Å for all Cα atoms and 1.1±0.6 Å for local, interlock-defining Cα atoms (Table S17). This is because LassoPred tends to select residues near the correct plug.

Compared to the ring region, the loop and tail region contribute more to the overall conformational uncertainty (1.2 Å for ring, 1.4 Å for loop, and 1.3 Å for tail, Table S18). This is consistent with the observation that LaPs with a shorter tail give a smaller RMSD value (e.g.,

RMSD for $^{test}$V1: 2.6 Å, $^{test}$V5: 1.2 Å, and $^{test}$V10: 3.1 Å). In contrast, $^{test}$V3 (4.8 Å) and $^{test}$V4 (4.4 Å) perform poorly due to their long and linear tails. With a 30 ns MD sampling, the linear tail artifact can be partially removed and will not cause the thread to move or unthread. For example, the RMSD value for xanthomonins I ($^{test}$V4) drops to 3.1 Å based on the representative structure from conformational clustering (from 4.4 Å in the optimized structure) (Figure 3A). Besides the test set, we applied LassoPred to build 3D structures for 5 uncharacterized LaPs shown in Figure 1E. LassoPred folds all LaPs into a lariat-like structure and the C-terminus threading into the ring (only the top predicted structure is shown, Figure 3B). Unlike AlphaFold3, which does not have the functionality to form the isopeptide bond, LassoPred ensures that all $^{iso}$C---$^{iso}$N distances are within the covalent bond regime (<1.4 Å).

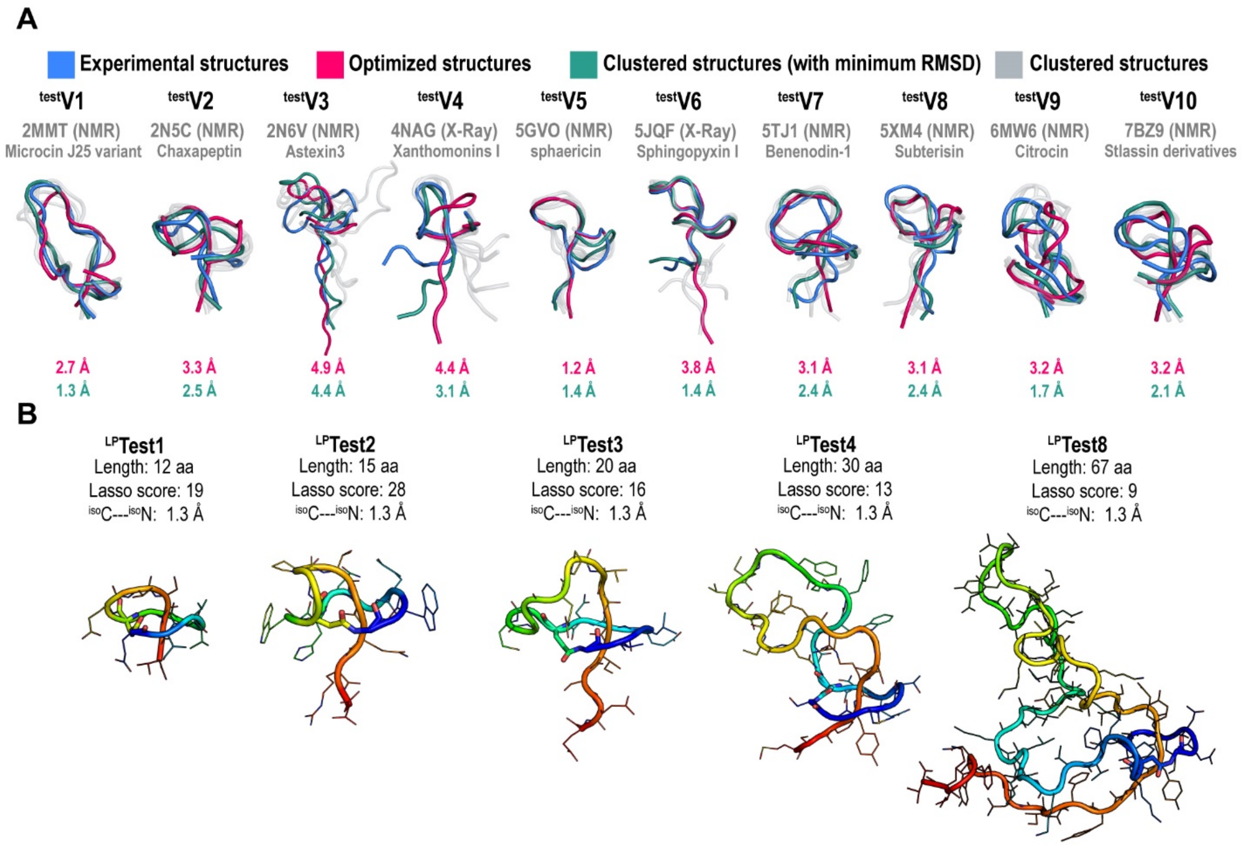

**Figure 3.** Assessment of LassoPred's constructor. (A) Prediction of LaP structures in the test set from the selected split (Table S11). All but $^{test}V_7$ have correct ring/loop/tail annotations predicted within their top 3 predictions. The illustrated structures correspond to the correct annotation for all LaPs except $^{test}V_7$. For $^{test}V_7$, the structure shown reflects the top prediction rather than the correct annotation. The 5 clustered structures (in gray) from 30 ns MD simulations and optimized structures (in pink) were compared with the experimental structures (in blue), with the minimum RMSD clustered structure highlighted in green and the RMSD value at the bottom. (B) Predicted structures for lasso peptide sequences using LassoPred. The structure ribbon is colored from blue at the N-terminal to red at the C-terminal. The lasso peptide sequences are the same as those shown in Figure 1E. Isopeptide C---N distances were measured between the N atom of the first residue and the C atom of the carboxyl group in isopeptide residues. The sequences for the tested LaPs are available in Table S4.

We further assessed the constructor's performance on 47 known LaP PDB structures, assuming correct sequence annotations for each. The results indicate that LassoPred-generated structures involve an average RMSD of 3.4±1.9 Å (Table 2). After MD sampling, the average RMSD of 47 PDB structures drops to 3.0±1.4 Å for the 30 ns trajectory and 3.0±1.3 Å for the 100 ns trajectory (Table 2), with a high global distance test total score (GDT-TS)[39] of 0.8. These results suggest that LassoPred is capable of generating accurate 3D structures, even without extensive MD sampling. To further assist users, we provide the option to generate MD sampling scripts for their LaP structures, allowing for additional refinement using local computing resources. These predicted structures can serve as starting points for downstream applications, such as design of molecular switches [13, 14, 40], docking for enzyme inhibitors[3, 41], and extensive sampling of LaP folding landscapes [42-45].

**Table 2.** Performance evaluation of LassoPred's constructor among 47 PDB structures, assuming correct sequence annotation for each. Average RMSD (given in Ångströms) and global distance test total scores (GDT-TSs), with standard deviation for LassoPred are computed across minimized and clustered structures, with all measurements using the experimental structures as reference.

| | **RMSD** | **RMSD (ring)** | **RMSD (loop)** | **RMSD (tail)** | **RMSD (ring+loop)** | **GDT-TS-score** |
|---|---|---|---|---|---|---|
| LassoPred optimized | 3.4±1.9 | 1.1±0.7 | 1.4±1.4 | 1.7±1.8 | 2.0±1.5 | 0.7±0.1 |
| MD clustered (30 ns) | 3.0±1.4 | 1.1±0.4 | 1.4±1.2 | 1.3±1.2 | 2.1±1.2 | 0.8±0.1 |
| MD clustered (100 ns) | 3.0±1.3 | 1.1±0.4 | 1.4±1.2 | 1.2±1.2 | 2.1±1.0 | 0.7±0.1 |

#### 2.4 Lasso Peptide Structure Prediction and Database Web App

Leveraging LassoPred, we predicted 3D structures for lasso peptides with an undetermined structure. Using the 4,749 unique LaP sequences previously identified by RODEO genome mining [15, 16] (Supporting Information, Data.csv), we applied LassoPred to create an optimized structure for each predicted sequence annotation, yielding 13,866 LaP structures. Compared to the 47 existing PDB structures, which only include LaPs from three phyla (pseudomonadota: 59.6%, actinomycetota: 38.3%, and bacillota: 2.1%, Figure 4A), the newly predicted structures from LassoPred increase phylogenetic diversity, spanning 21 phyla (Figure 4B). These include 38.8% from pseudomonadota, 32.6% from actinomycetota, 15.7% from bacillota, and smaller proportions (each below 10%) from cyanobacteriota, bacteroidota, euryarchaeota, and others (Figure 4C). Furthermore, the sequence length of these predicted structures ranges from 12 to 160 aa residues, significantly broader than the 14-33 aa residue range in existing PDB structures, representing a

7.8-fold increase in range expansion (Figure 4D). The loop and tail length also expands to a range of 3-50 and 2-51 aa residues, respectively (Figure 4D). To highlight the sharp difference in structural scope, Figure 4E displays relaxed LaP structures with the long tail length (i.e., 51 aa, LP_QOR62253_3) and maximum loop length (i.e., 50 aa, LP_EDM37169_1). All these structures represent the most populated conformational cluster from a 30 ns MD production (Figure 4E and 4F). We observed helical secondary structures in the notably extended regions of the structures, specifically in the tail region of the max-tail structure (positions 60-66, stabilized by $^{Gly63}$N---$^{Thr66}$O hydrogen bond at 3.0 Å) and in the loop region of the max-loop structure (positions 45-48, stabilized by $^{Arg45}$N---$^{Leu48}$O hydrogen bond at 3.5 Å). These more globular domains have not been observed in existing lasso peptide structures, raising the interesting possibility that the ring and tail of lasso peptides are used as stabilization motifs for larger globular protein domains.

To allow public access, we developed a web interface for LassoPred, including a database and a prediction tool (Figure 4G). The database, containing 13,866 optimized structures from 4,749 LaP sequences, enables users to conduct comprehensive searches based on major characteristics such as peptidase ID, phylum, precursor, leader, and core sequences. For each entry, a prediction summary provides up to top three rankings of ring and loop length pairs, along with the optimized structure, any available relaxed structures, and MD simulation files for each rank. The prediction tool allows users to submit tasks for predicting 3D lasso peptide structures from an input sequence, download results from our server, and receive updates via email. The result files include content similar to each database entry. Although no production MD simulations will be run on our server, the users will receive essential input files to initiate a MD simulation. The whole process is expected to take less than 10 minutes. In summary, LassoPred provides an accessible and comprehensive lasso peptide structure prediction tool and database, assisting in the discovery of functional lasso peptides.

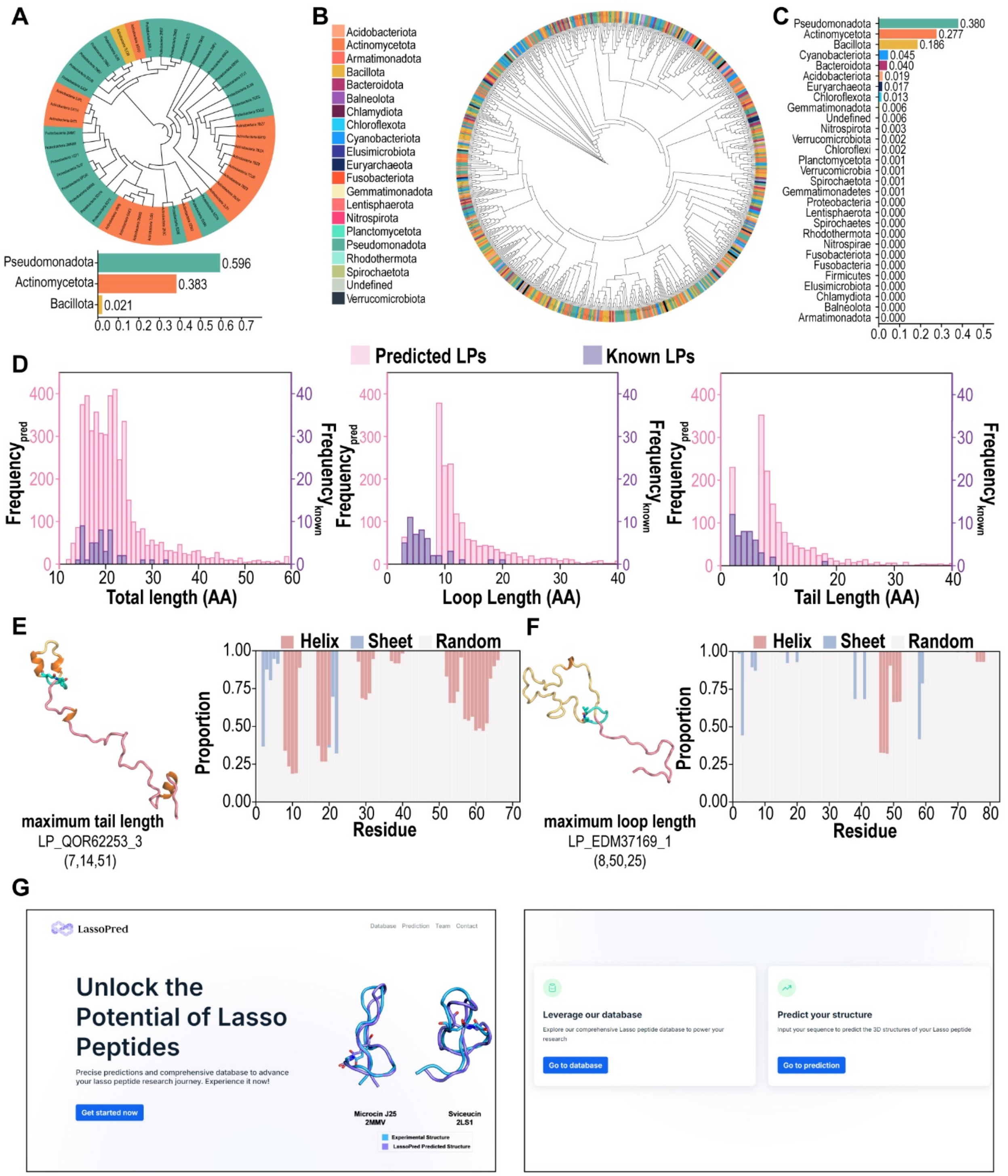

**Figure 4**. Database and prediction tools for lasso peptide structures. (A) Phylogenetic tree constructed from the amino acid sequences of 47 existing lasso peptide structures. Colors are assigned according to their phylum, with the proportion of each phylum displayed at the bottom. (B) Phylogenetic tree of 4,749 unique known lasso peptide sequences, which were first clustered to 680 sequences and then aligned to construct the tree; labels are color-coded by phylum. (C) Phylum distribution of 4,129 unique known lasso peptide sequences in the database, using the same color scheme as in (B). (D) Comparison of total length, loop length, and tail length between existing and predicted LaPs. Lengths exceeding 60 are omitted due to their scarcity. (E) Representative structure from the database with max tail and (F) max loop, along with their respective proportions of secondary structures. The clustered structures from 30 ns MD were considered as representative structures. Each structure is labeled by its database ID, with ring, loop, and tail lengths noted in brackets. (G) Representative features of the LassoPred web interface.

### 3. Discussion

LassoPred and its associated database have a potential to advance fundamental knowledge about lasso peptides, accelerate the discovery of new functional peptides, and inform the design of new tools to aid in lasso peptide design and engineering, demonstrating a huge potential for shifting the paradigm of lasso peptide research. The database expands the number of LaP structures from 47 to 4,749. We acknowledge that the training dataset comprised only 47 lasso peptides, reflecting the relative scarcity of experimentally characterized LaP structures in the field. To date the majority of lasso peptide structures have been solved using solution NMR techniques with a smaller number of structures determined by X-ray crystallography. Regardless of the technique, a campaign to determine a lasso peptide structure takes a minimum of several months of experimental work (sometimes extending to years). Moreover, success is not guaranteed; we have published our unsuccessful attempts at solving the structures of cellulonodin-2[26] and fuscanodin [25], also known as fusilassin[46]. We have attempted experimental validation of LassoPred by trying to solve the NMR structure of a novel lasso peptide that harbors antimicrobial activity. Despite the fact that this peptide expressed well in a heterologous host, the 2D NMR experiments did not yield data of a quality sufficient to determine the structure after varying solvents, the temperature of acquisition, and NOE mixing times.

These challenges inherent in lasso peptide structure determination highlight a critical gap that LassoPred fills by providing large-scale in silico structural predictions beyond the small set of known structures. These predicted structures can be used in docking simulations or other *in silico* drug discovery efforts. These predictions provide basis to elucidate the sequence-structure-function relationship underlying the  extraordinary thermostability of LaPs and stability in solvents other than water [14, 47], inspiring the development of rational engineering strategies to tune lasso peptide properties [48]. The construction of lasso peptides with a non-native left-handed wrapping fold, as enabled by LassoPred, facilitates researchers to investigate the folding landscape of lasso peptides [43, 45] and the origin of lasso peptide wrapping handedness, thereby enhancing the fundamental knowledge about lasso peptides.

LassoPred can aid in prioritizing new LaPs for discovery as antibiotics and self-assembled biomaterials. Notably, LaPs like microcin J25 and capistruin are potent RNA polymerase (RNAP) inhibitors by binding to the RNAP secondary channel, blocking access of nucleotides to the RNAP active site, as well altering the folding of loops that are essential for RNAP's catalytic activity [8, 49]. While sequence patterns from known antimicrobial lasso peptides, such as the "two-tyrosine" motif, have been used alongside bioinformatics to guide the discovery of new lasso peptide antibiotics[50, 51], structure-based molecular modeling—docking and free energy perturbation—enables the *in silico* discovery of lasso peptides targeting other cellular components, such as cell wall synthase, ribosome[52], membrane transporters [53]. This could lead to new antimicrobial

LaPs for treating life-threatening Gram-negative bacteria. The LassoPred database also allows researchers to locate new types of interlocked rotaxane switches like benenodin-1 [13, 54], and cysteine-containing lasso peptides as dynamic covalently-bonded, self-assembled biomaterials [12] for medical and industrial uses [55]. Besides boosting the *in silico* discovery of functional LaPs, the LassoPred database hosts thousands of sequences and structures for building and optimizing predictive machine learning model, such as DeepLasso [56]. These data can also facilitate the construction of generative models to achieve *de novo* design of lasso peptides.

In the end, we would like to discuss the technical limitations of LassoPred and how these issues can be potentially mitigated through joint computational and experimental efforts. First, one limiting factor of LassoPred's performance lies in its moderate top 1 accuracy (i.e., ~60%). Although this accuracy value is significantly higher than the baseline probability of randomly guessing the correct plug position in an average-length lasso peptide (~11%, estimated using microcin J25, see Text S4), there is still room for improvement in the model's predictive performance.

Second, LassoPred is unable to accurately predict the temperature-dependent behavior of lasso peptides, a common limitation among current protein structure prediction tools. For example, in the case of benenodin-1, which adopts multiple isomeric states at higher temperatures, LassoPred can identify isomeric state 2 (PDB ID: 6B5W, loop/tail length: 7/4) and a minor conformational state (loop/tail length: 5/6) within its top 3 predictions [57]. However, it is unable to predict changes in the population of these states at different temperatures. A viable solution to this issue is to employ multiscale molecular simulations, integrating quantum chemistry-based energy modeling with conformational entropy calculations [42].

Third, LassoPred may generate lariat knot-like folds for any input lasso-like sequence, potentially leading to false positives. To prevent this, we have used valid LaP sequences from RODEO that assesses the likelihood of a sequence to be a true lasso peptide by considering its neighboring sequences of leader peptide, lead peptide binding proteins, transporters, leader peptide hydrolases, lasso peptide cyclases, and isopeptide hydrolases. In addition, as *in vitro* lasso peptide construction[17, 25] and cyclase engineering technologies[58] advance, most components of lasso peptides will become mutable, ultimately achieving the long-term goal of converting any arbitrary lasso-like sequence into a lasso peptide.

Fourth, the current version of LassoPred does not consider a diverse range of structural scaffolds caused by disulfide bond formation. Among the 3,131 LaPs, we observed 226 LaPs containing 2 Cys, 34 containing 4 Cys, 7 containing 6 Cys. Though occupying less than 10%, the disulfide bond-containing LaPs typically involve a high thermostability and potential to form mechanically interlocked structures and materials. The 7 LaPs with 6 Cys will also extend to a new class of LaP beyond the known types (up to 4 Cys in existing LaP structures, Table S22). Based on the structures of these 7 LaPs built by LassoPred, we will develop new LassoPred functions to construct all possible disulfide bond-containing LaPs (see Figure S6 as an example). These structures will lead to new hypotheses for experimentally characterizing and understanding new classes of LaPs. Specifically, whether these disulfide linkages originate from intrinsic conformational distribution, or from its interactions with enzymes during its biosynthesis.

Last but not the least, new modules should be developed to enhance LassoPred's discovery capabilities, including a docking module to inform how LaPs interact with cellular protein targets for drug discovery, an enhanced conformational sampling and Markov State Model analysis module to elucidate the key conformational populations underlying LaPs' functions, artificial intelligence-based scoring functions to predict the impact of mutation on LaPs' physical and pharmaceutical functions, and the option to incorporate 3D visualization of lasso peptide structures on the website.

### 4. Computational Methods

**4.1 Data Curation for the Training and Test Dataset.** We collected all known structures of lasso peptides from the Protein Data Bank (PDB)[59] (accessed on 04-01-2024), compiling a dataset of 50 lasso peptide structures (see Tabel S5). To prevent data leakage, we removed entries with identical sequences and annotations, resulting in 47 sequences. We manually annotated the isopeptide-donating residue, upper plug, lower plug, ring length, loop length, and tail length on each lasso peptide structure (see definition in Figure S1). The resulting dataset comprises sequences and annotations for 47 lasso peptides (Table S5).

**4.2 LassoPred's Annotator to Annotate Sequence Regions.** LassoPred's annotator employs two machine learning classifiers, isopeptide classifier and plug classifier, to pinpoint the location of the isopeptide ($N_{iso}$) and upper plug residue ($N_{up}$), respectively, thereby deriving the length of the ring ($N_{iso}$), loop ($N_{up}– N_{iso}$), and tail ($N–N_{iso}$) for a LaP sequence with N amino acids.

*Sequence Fragmentation.* Both sub-classifiers were trained and tested using data generated by splitting LaP sequences into consecutive dipeptide fragments (see benchmarks on fragmentation strategies, Table S7-S9), where each fragment overlaps with its neighboring fragments by two amino acids. To ensure that the C-terminus residue can be labeled as the "tail" fragment, one dummy amino acids (denoted as "B") are appended. As such, each LaP sequence generates N fragments, leading to a total of 905 dipeptide fragments from 47 PDB-curated sequences. Each fragment is labeled separately for both sub-classifiers. For the isopeptide classifier, a dipeptide is labeled as 1 if all two residues reside in the ring, 0 if they reside at the boundary between the ring and loop (i.e., contains the isopeptide-donating residue), and 2 if they reside in the loop or tail. For the plug classifier, a dipeptide is labeled as 1 if all two residues reside in the ring or loop, 0 if they reside at the boundary between the loop and tail (i.e., contains the upper plug and lower plug), and 2 if they reside in the tail. The terminology "plug residue" do not have implication on their mechanistic roles in holding the ring in place. The "plug position", annotated as "0", indicates the two residues between which the ring is located based on the PDB structure, rather than which two are mechanically locking the peptide. We applied separate truncation strategies to avoid noise for both sub-classifiers: for the isopeptide classifier, the $6^{th}$–$10^{th}$ amino acids are retained, while for the plug classifier, the sequence region for the loop and tail is kept. Both classifiers were trained separately to predict the probability of observing the isopeptide bond and upper plug residue within each dipeptide, thereby informing the location of the isopeptide ($N_{iso}$) and upper plug residue ($N_{up}$) for sequence annotation.

*Repeated holdout validation*. To enhance the model's robustness and reduce the impact of the small dataset on generalizability, we employed the repeated holdout validation to evaluate the model performance and to select model-building strategies and hyperparameters [34]. We conducted 100 training/test set splits at a 4:1 ratio with stratified sampling to ensure that the test set maintains a consistent distribution of ring and loop lengths with the overall dataset (splits_100.csv). Machine learning algorithms and their hyperparameters were selected based on the split with the minimum absolute error from the mean across various performance metrics, including fragment classification accuracy, fragment classification F1 score, one-vs-rest weighted ROC AUC, ROC AUC for Class 0, Class 1, and Class 2, as well as top 1, top 2, and top 3 accuracy (Table S11). To prevent multivalued mapping, the two isomeric states of benenodin-1 (PDB ID:

5TJ1 and 6B5W) [13] were placed in separate datasets (training vs. test). With benchmarking on fragmentation (Table S7-S9) and featurization (Table S10) strategies, the selected data set were determined based on the MAE on both isopeptide and plug predictors (Table S11). The final dataset contains 148 dipeptides for training and 40 for testing in the isopeptide classifier, and 409 dipeptides for training and 103 for testing in the plug classifier.

*Classifier Training and Testing*. We trained the model on 37 sequences and tested it on 10 sequences. For featurization, we used the ESM2 language model to represent lasso peptide sequences, using the output from Layer 33 of the "ESM2_L33_650M" model, which produces features of dimension 1280 corresponding to the sequence length. Notably, the same dipeptide fragment from different sequences is assigned with a different feature because ESM2 considers both the overall sequence context and the specific amino acids within that sequence. Additional feature benchmarks are provided in the SI (Table S10). Using ESM2 embedding features and the final selected dataset, we benchmarked various models, including K-Nearest Neighbors[60], SVC[61], Random Forest[62], and Gradient Boosting Classifier[63], assessing each model by its accuracy, F1 score, and ROC AUC[64]. This was achieved by searching over predefined parameter grids for each model by GridSearchCV from scikit-learn [65] (Table S6). Support vector classifier was selected for validation on the test set (10 held-out sequences) due to its best-performing ROC AUC for both the isopeptide and upper plug predictors. The optimized hyperparameters include the regularization parameter C (0.1), degree of the polynomial kernel function (3), gamma ('scale'), kernel type ('linear'), random state (42).

*Sequence Region Annotation*. For each LaP sequence with unknown ring, loop, and tail length, LassoPred's annotator employs its classifiers to infer the location of the isopeptide ($N_{iso}$) and upper plug residue ($N_{up}$), printing out one $N_{iso}$ value and three $N_{up}$ values. LassoPred first employs the isopeptide bond predictor to access the probability of each constituent dipeptide fragment containing the isopeptide-donating residue (labeled as “0”, described in the *Sequence Fragmentation* section). LassoPred then calculates the probability of dipeptide fragment being labeled as “0”. Other probability inference strategies are benchmarked in the SI (Table S8-S9). The presence of “0” for residue $N_x$ and $N_x$+1 in a dipeptide fragment indicates a potential isopeptide position at $N_x$+1. LassoPred determines the isopeptide position to occur in the highest-ranked “0” dipeptide whose residue $N_x$+1 is Asp or Glu, located in the $7^{th}$, $8^{th}$, or $9^{th}$ amino acid position. Similar to the approach of determining $N_{iso}$, LassoPred employs the upper plug predictor to access the probability of dipeptide fragment containing the upper plug (labeled as “0”), and rank the probability of fragments with “0” labeling. LassoPred predicts three potential upper plug positions ($N_{up}$) from top-ranking residue pairs that occur in the sequence range of [$N_{iso}$+3, N-2]. This range is determined based on the empirical observation that existing LaPs involve minimum loop length of 3 and a minimum tail length of 2. Consequently, with one input LaP sequence, LassoPred predicts three possible sets of sequence annotations.

**4.3 LassoPred’s Constructor to Build 3D Structures.** LassoPred’s constructor upgrades LassoHTP[33] by generating structures faster, using a larger scaffold library, enabling construction of left-handed structure, and performing MD clustering. The constructor comprises three modules: scaffold construction, mutant generation, and an optimization. These work together to build 3D lasso peptide structures with predicted sequence annotations (ring, length, and loop length). The scaffold construction uses an expanded scaffold library as initial backbone for ring and loop regions, and use PyMOL’s ‘fab’ function to build tail region[66]. We used SWISS-MODEL[37] to generate additional lasso peptide scaffolds based on known right-handed structures, reducing the time for each scaffold construction from 2 hours to 10 minutes compared to LassoHTP. We varied the loop length from 3 to 50 residues for ring sizes of 7, 8, or 9 by inserting alanines. All residues were mutated to glycine to eliminate chirality, and the tail was truncated. Left-handed scaffolds were obtained by mirroring the right-handed ones using PyMOL. The mutant generation module then modified the scaffold residues according to the input sequence using PyMOL. Finally, the optimization module optimizes the structure using AMBER22 pmemd.cuda[67], providing optional MD simulations and clustering analysis.

*Molecular Mechanics Modeling*. We generated force field parameters for isopeptide bonds involving non-standard residues (Glu or Asp) through a two-step restrained electrostatic potential (RESP) charge fitting approach[68]. Using the Antechamber package with the Generalized Amber Force Field (GAFF) parameters[69], we set the bond, angle, dihedral, and van der Waals parameters for these peptides. We constructed the peptide force field using ff14SB[70], and solvated the peptide in TIP3P water molecules [71]using an octahedral box with a 10.0 Å buffer. Sodium or chloride ions were added to neutralize the system. The MD simulation protocol starts with energy minimization with up to 10,000 cycles, employing a 10.0 Å non-bonded interaction cutoff and applying harmonic positional restraints of 20 kcal/mol·Å$^2$ to the $C_\alpha$ atoms. Long-range electrostatics was treated using the Particle Mesh Ewald method. SHAKE algorithm was applied to constrain bonds and angles involving hydrogen atoms [72, 73]. The system was heated to 300 K over 40 picoseconds using a Langevin thermostat[74] and a Berendsen barostat[75], with restraints on the $C_\alpha$, N, and C atoms; equilibrated for 1 ns with NPT ensemble at 300 K and 1 atm, using restraints on backbone atoms; equilibrated for 1 ns NVT equilibration with no restraints; and eventually underwent a 30 ns NPT production with a 2 fs time step at 300 K and 1 atm. The resulting MD trajectory was clustered to identify 5 representative structures (Text S2). We performed root-mean-square deviation (RMSD), hydrogen bond assessment, and radius of gyration ($R_g$) analysis using cpptraj[76]. Visualization of the structures was performed using PyMOL[66].

**4.4 Genome Mining and Database Construction.** The lasso peptide sequences were collected from previous genome mining research[15-17]. They obtained these sequences either from genomic data analyses using tools such as RODEO and RRE-Finder, or from experimental validations involving evolutionary covariance and biochemical assays by March 15th, 2024, yielding 1315, 5193, and 7701 LaPs, respectively. Notably, RODEO-derived sequences (n=1315) are high-confidence as they include complete biosynthetic gene clusters, whereas RRE-Finder predictions (n=5193) often lack supporting genes, and cell-free derived sequences (n=7701) may be foldable but non-functional. We cleaned the data by deleting sequences with completely consistent core sequences. We filtered the validated sequences by removing duplicates (reducing the total from 14,209 to 5,686 LaPs), excluding sequences where the 7th, 8th, or 9th amino acids are not capable of forming isopeptide bonds (i.e., not Asp or Glu), sequences with uncertain amino acids such as "X", sequences too short to meet the minimum loop (3 AAs) and tail length (2 AAs) requirements, and sequences too long to predict accurately (predicted loop length exceeding 57 residues). This process resulted in a curated set of 4,749 available sequences. We developed a database for known lasso peptides, to facilitate analysis of lasso peptides structures and mining of antimicrobial peptides. Each sequence characterized by attributes such as Lasso Peptide Family, Name, Query GI, Peptidase, Phylum, Leader Sequence, Core Sequence, and other sequence information like Sequence Length and Calculated Mass. The database's structure table hosts 13,866 optimized structures with unique core sequences predicted by LassoPred. This table provides up to three predicted structures per sequence, along with their MD simulation files (*.prmtop, *.inpcrd, *.in). The initial structure was optimized with the minimization (min*.pdb).

The database is accessible to the public via website interface (https://lassopred.accre.vanderbilt.edu/). We will update any changes to access instructions on this page in the future.

ASSOCIATED CONTENT

**Supporting Information**. Supplementary Figures 1–6, Text 1–4 and Tables 1–22.

Supplementary Data 1: Data.csv (curated and deduplicated lasso peptides in database).

Supplementary Data 2: splits_100.csv (100 test set splits at a 4:1 ratio with stratified sampling).

**Data Availability**. The lasso peptide structures from the dataset are available on GitHub (https://github.com/ChemBioHTP/LassoPred) and can also be downloaded from the Protein Data Bank (PDB) (https://www.rcsb.org/) using the PDB IDs listed in Table S4. The lasso peptide sequence database is available from ref. [16] and ref.[15]. All predicted LaP structures can be downloaded from LassoPred database: https://lassopred.accre.vanderbilt.edu/.

**Code Availability**. The LassoPred software, including detailed tutorial, is freely available on GitHub (https://github.com/ChemBioHTP/LassoPred). The source code is also available via Zenodo at https://doi.org/10.5281/zenodo.11619636.

AUTHOR INFORMATION

**Corresponding Author**

*Email: zhongyue.yang@vanderbilt.edu phone: 615-343-9849

*Email: ajlink@princeton.edu phone: 609-258-7191

*Email: yileizhao@sjtu.edu.cn

**Notes**

The authors declare no competing financial interest.

ACKNOWLEDGMENT

This work was supported by the startup grant from Vanderbilt University, the fellowship of Vanderbilt Institute of Chemical Biology, and the sponsorship from Rosetta Commons Seed Grant Award. X.O. and Y.-L.Z. acknowledge the grant of National Key R&D Program of China (2020YFA0907700, 2018YFA0901200), and the grants from National Science Foundation of China (31970041). A.J.L. acknowledges NIH grant GM107036. This work leveraged the Research Software Engineer Services provided by the Vanderbilt Advanced Computing Center for Research and Education (ACCRE), a collaboratory operated by and for Vanderbilt faculty.

CONTRIBUTIONS

X.O. developed the machine learning models, collected the training and test data, built the structure construction pipeline, and wrote the paper. X.R. designed the sequence fragmentation strategy and machine learning models, and deployed the website interface. H.X. designed the website UI/UX. Y.-L.Z. and AJL supervised the study and revised the paper. Z.J.Y. designed and supervised the study and wrote the paper.

## Supplementary Files

This is a list of supplementary files associated with this preprint. Click to download.

- splits100.csv
- Data.csv
- SI.pdf
